# Developing Strategies to Treat Asthma Exacerbations Caused by Rhinovirus

**DOI:** 10.1016/j.ebiom.2014.12.012

**Published:** 2014-12-31

**Authors:** Peter W. Heymann

**Affiliations:** University of Virginia Asthma and Allergic Diseases Center, Department of Pediatrics, P.O. Box 800386, Charlottesville, VA 22908-0386, United States

Rhinovirus (RV) accounts for 75 to 80% of virus-induced exacerbations of asthma among children and young adults. A wide diversity of RV genotypes cause recurrent infections that lead to attacks of wheezing that begin in infancy. After 3 years of age, it is now clear that atopy and allergic airway inflammation are major risk factors for attacks of asthma provoked by RV (Heymann et al., 2004, Soto-Quiros et al., 2012). Together with efforts to understanding mechanisms underlying the role of RV in causing asthma exacerbations, current research has been focused on determining whether there are strains of RV that are more likely to induce an attack of asthma to guide the development of new treatments, including vaccines.

A significant challenge for developing vaccines against RV is that well over 150 strains of RV have been identified. These strains belong to three genetically related groups; RV-A, B, and C. Recent investigations indicate that some strains may be more likely to provoke an asthma attack and may represent targets for an effective vaccine. In population-based studies of children and adults experiencing an attack of asthma, group A and C strains have been detected more frequently. Recent studies from Australia and Costa Rica indicate that the group C strains may trigger exacerbations more often than group A strains (Soto-Quiros et al., 2012, Bizzintino et al., 2011); however, studies from the United States indicate that the group A viruses play an equally important role (Khetsuriani et al., 2008, Kennedy et al., 2014).

Deciphering the pathogenic role of each RV strain in provoking asthma symptoms has also been challenging. PCR tests for RV are frequently positive in longitudinal studies designed to determine the rate of infections (e.g., approximately 0.4 to 0.6 infections/month in children) (Winther et al., 2006). However, many of these positive tests either represent sub-clinical infections, or identify recent, but not acute infections. Unfortunately, cultures underestimate the prevalence of RV infections, because the group C strains cannot be detected using currently available culture systems. For these reasons, the research paper in this issue of *EBioMedicine* by Niespodziana and colleagues addresses a very important problem (Niespodziana et al., 2015--in press). Their paper describes the measurement RV group and strain specific antibody responses to recombinant RV related proteins and fragments. The results indicate that IgG1 isotype antibody responses specific for the N-terminal fragments of the VP1 coat protein may serve as serologic markers for clinically relevant RV infections associated with asthma exacerbations that might be valuable in future epidemiologic studies (Niespodziana et al., 2015--in press).

More specifically, the paper by Niespodziana et al. describes the production of recombinant RV coat proteins (VP1–4), together with nonstructural replication proteins, and the major epitope containing N-terminal fragments of VP1 proteins derived from 4 RV strains (including two strains of group A RV's, and one strain each from the group B and group C RV's) (Niespodziana et al., 2015--in press). This is a significant achievement which allowed the investigators to evaluate antibody isotype responses to these recombinant reagents by ELISA. The assessments were done using sera obtained prior to and following the inoculation of subjects with RV-16, a group A strain. The subjects included those with mild asthma, moderate asthma, and healthy, non-atopic controls. The results revealed that the experimental infection with RV-16 induced a strain specific boost of antibody production by day 42 (6 weeks post-inoculation), predominantly against the N-terminal epitope on VP1. This response was stronger among the subjects with moderate asthma and correlated with the severity of upper and lower respiratory tract symptoms (Niespodziana et al., 2015--in press).

To learn whether measurements of IgG1 to the N-terminal portion of VP1 coat proteins might be useful as serologic tests for the most common, clinically relevant strains of RV, the authors note that further work is needed. For example, comparative sequence modeling predicts that all RV-C VP1 proteins are shorter by 21 residues compared to VP1 proteins from RV-A strains (Basta et al., 2014). Additionally, IgG1 antibody titers to RV-C VP1 proteins recently were shown to be reduced compared to titers directed against RV-A and B strains in plasma samples from children presenting to the ER with asthma exacerbations (Iwasaki et al., 2014). Of course, the timing of sample collection may be critical for evaluating these responses. Unfortunately, a group C strain of RV is not yet available for experimental challenge, but the authors point out that it will be important to obtain more data regarding the immunogenicity of RV-C VP1 proteins, especially the N-terminal fragments, to gain more information relevant to vaccine development. Also interesting is that IgG1 antibody to the N-terminal VP1 fragments from RV groups A, B, and C was detectable before virus inoculation, followed by a significant group A strain specific boost by day 42 after inoculation. Not known is how long these IgG1 titers would remain elevated post-infection which could confound efforts to identify a strain linked to a subsequent RV-induced asthma attack, especially among children who get RV infections more frequently.

Looking to the future, the development of effective anti-RV vaccines may be further complicated by other picornavirus strains that can cause asthma attacks, including enterovirus-68 which recently caused hospital admissions and ER visits for asthma in the U.S. this fall. Thus, other treatment strategies remain under investigation and include the development of innate anti-viral therapies (e.g., administering type I or type III interferons); however, costs, side effects, and how these drugs should be administered (seasonally, or at the time of an acute infection) will need to be considered. Alternatively, treatments to reduce allergic inflammation in the airway may also help to counter the asthmatic effects of RV. In support of this approach, the administration of omalizumab (anti-IgE antibody) to inner-city children with asthma in the US virtually illuminated the seasonal increases in asthma exacerbations in the spring and fall (Busse et al., 2011). This treatment effect included a subset of children who tested positive for RV. As these treatment strategies are compared and evaluated, the research results described by Niespodziana et al. clearly represent a step in the right direction to help us develop new methods to minimize the adverse effects of RV infections in the asthmatic population.

## Conflicts of Interest

The author declares no conflicts of interest.

## Funding Support

The author is supported by the National Institutes of Health Grants: U01-AI100799 and R01-AI020565.
